# Predicting ketosis during the transition period in Holstein Friesian cows using hematological and serum biochemical parameters on the calving date

**DOI:** 10.1038/s41598-022-04893-w

**Published:** 2022-01-17

**Authors:** Seungmin Ha, Seogjin Kang, Manhye Han, Jihwan Lee, Hakjae Chung, Sang-Ik Oh, Suhee Kim, Jinho Park

**Affiliations:** 1 National Institute of Animal Science, Rural Development Administration, Cheonan, Republic of Korea; 2 Department of Internal Medicine and Biomedical Research Institute, Gyeongsang National University Hospital, Jinju, Republic of Korea; 3 College of Veterinary Medicine, Chonbuk National University, Iksan, Republic of Korea

**Keywords:** Diseases, Health care

## Abstract

Ketosis often occurs during the postpartum transition period in dairy cows, leading to economic and welfare problems. Previously, ketosis was reported to be associated with hematological and serum biochemical parameters. However, the association between the parameters on the calving date and ketosis during the postpartum transition period remains unclear. This study aimed to investigate this association. Blood samples were collected from the jugular vein of Holstein cows on the calving date and β-hydroxybutyrate was tested once every 3 days (8 times in 21 days). The cows were divided into three groups: non-ketosis, subclinical ketosis, and clinical ketosis. The clinical ketosis group significantly had the highest values of mean corpuscular volume, mean corpuscular hemoglobin, β-hydroxybutyrate, non-esterified fatty acids, and total bilirubin, but the lowest values of red cell distribution width, the counts of white blood cell, monocyte, and eosinophil, albumin, alanine transaminase, lactate dehydrogenase, and amylase. In contrast, the non-ketosis group showed the opposite results (*p* < 0.05). In conclusion, these parameters are associated with the development and severity of ketosis. Our findings suggest that these parameters on the calving date may be useful indicators to identify dairy Holstein cow susceptible to ketosis during the transition period.

## Introduction

Ketosis, a phenomenon associated with negative energy balance (NEB), is classified into three types: type I (spontaneous or underfeeding ketosis), type II (fatty liver), and butyric acid silage ketosis. Type II ketosis occurs in the postpartum transition period^1,2^. All dairy cows experience NEB, since in early lactation there is a higher energy requirement for milk production than the energy intake by feed^3,4^. However, NEB does not necessarily lead to ketosis in all dairy cows. The occurrence of ketosis depends on whether cows overcome NEB through metabolic adaptation.

Ketosis causes economic and animal welfare concerns in the dairy farm industry. Numerous studies have reported factors associated with ketosis in dairy cows including body condition score, breed, age at first calving, cow parity, calving season, dystocia, calving interval, dry period, prolonged previous lactation length, herd size, increased colostral production, milk protein percentage and 305-day milk yield and milk fat yield in the previous lactation, metritis, milk fever, retained placenta, and calf sex^5–11^. In addition, a previous study suggested that breeding values could be used to predict which Holstein cows are susceptible to ketosis^12^.

Hematological and serum biochemical parameters are widely used to evaluate and monitor health, and these parameters have been demonstrated to be associated with ketosis. For example, cows with severe ketosis displayed higher hematocrit and hemoglobin values, while mean corpuscular volume (MCV), mean corpuscular hemoglobin (MCH), mean corpuscular hemoglobin concentration (MCHC), and red cell distribution width (RDW) did not differ between non-ketotic and ketotic cows^13^; in addition, cows with ketosis had significantly lower white blood cell (WBC), neutrophil, and eosinophil counts than those without ketosis^14^, and impaired white blood cell functions were associated with ketosis in dairy cows^15^; moreover, cows with ketosis showed increased non-esterified fatty acids (NEFA), aspartate aminotransferase, and total bilirubin (TB) and decreased levels of glucose, blood urea nitrogen (BUN), total protein, albumin (ALB), and triglyceride (TG)^2,16–21^; furthermore, concentrations of minerals, such as calcium, phosphorus, copper, and zinc, were associated with ketosis as well^22–24^. However, to the best of our knowledge, no previous studies have reported the association of ketosis during the postpartum transition period with hematological and serum biochemical parameters on the calving date.

We hypothesized that dairy Holstein cows show different levels of hematological and serum biochemical parameters according to calving date before the incidence of ketosis. Accordingly, the objectives of this study were (1) to identify which hematological and serum biochemical parameters are associated with ketosis and its severity (non-ketosis, subclinical ketosis, and clinical ketosis), (2) to investigate how these parameters are associated with ketosis, and (3) ultimately, to offer the possibility to predict ketosis during the postpartum transition period by using these parameters on the calving date. The present findings would help to identify dairy cows at a high risk of ketosis and to establish a strategy to reduce ketosis damages.

## Results

### Descriptive statistics for the dairy cows by ketosis

According to the highest β-hydroxybutyrate (BHBA) concentration among eight measurements during the postpartum transition period, 126 Holstein Friesian cows were divided into three groups: non-ketosis (NK), subclinical ketosis (SCK), and clinical ketosis (CK). The NK group accounted for 50% (n = 63) of the population, while 26.98% (n = 34) and 23.02% (n = 29) of cows had SCK and CK, respectively. Both SCK and CK groups were most common on day 9. The parity, age, and body condition score were the highest in the CK group, whereas the NK group had the lowest parity, age, and body condition score. Moreover, the CK group produced the most milk, while the NK group produced the least milk in the early transition period, even though the differences of daily milk yield during the entire transition period were less than 10% (Table 1).

**Table 1:** Descriptive statistics for the Holstein Friesian cows included in the study.

	NK	SCK	CK
Number	63	34	29
Cow parity	2.0 ± 0.1	2.3 ± 0.2	2.6 ± 0.3
Age at calving, years	3.7 ± 0.2	4.6 ± 0.4	5.5 ± 0.5
Body condition score	3.00 ± 0.05	3.16 ± 0.08	3.36 ± 0.08
Daily milk yield (Day 4–Day 21), L	28.6 ± 0.5	31.3 ± 0.6	30.9 ± 0.7
Day 4–Day 6	23.0 ± 0.9	25.4 ± 1.1	27.4 ± 0.9
Day 7–Day 9	26.6 ± 1.0	28.9 ± 1.3	31.3 ± 1.7
Day 10–Day 12	28.8 ± 1.1	31.7 ± 1.4	30.8 ± 1.8
Day 13–Day 15	30.4 ± 1.1	32.9 ± 1.2	32.2 ± 1.7
Day 16–Day 18	30.6 ± 1.1	33.6 ± 1.2	31.2 ± 1.7
Day 19–Day 21	31.8 ± 1.1	34.5 ± 1.4	33.0 ± 1.9
**Incidence of ketosis by monitoring day**			
Day 0			
Day 3		3	2
Day 6		6	5
Day 9		8	10
Day 12		2	3
Day 15		5	5
Day 18		7	4
Day 21		3	

Data are expressed as mean ± standard error of the means.

NK, non-ketosis group; SCK, subclinical ketosis group; CK, clinical ketosis group. mean ± standard of error of the means.

### Association between ketosis and hematological parameters

**Figure 1:**
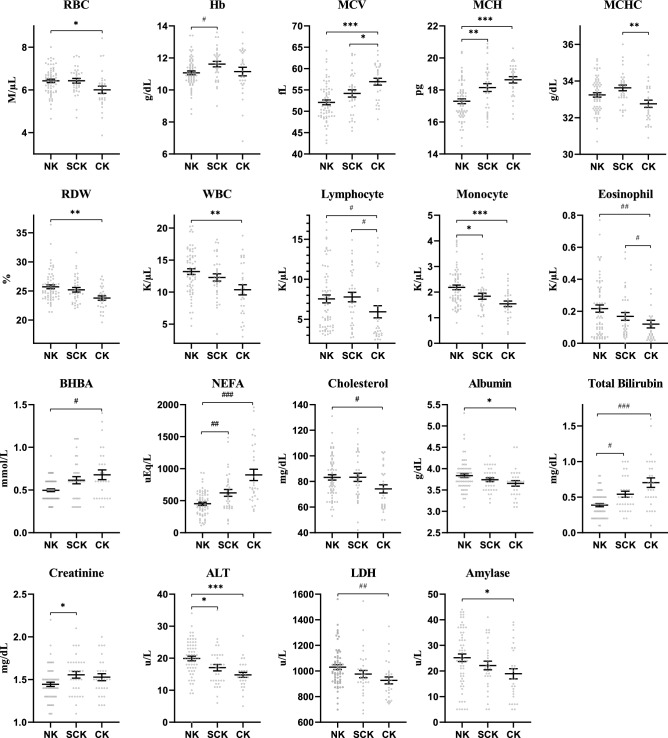
Hematological parameters including erythrocyte and leukocyte count and significant serum biochemical parameters. The results are expressed as mean ± standard error of the means. Statistical significance was expressed in one of the following two ways: one-way ANOVA with post-hoc Tukey honestly significant difference (*p < 0.05; **p < 0.01; ***p < 0.001) or Kruskal–Wallis test and Mann–Whitney U test with Bonferroni’s method (^#^*p* < 0.017; ^##^*p* < 0.003). ALT, alanine transaminase; BHBA, β-hydroxybutyrate; CK, clinical ketosis; Hb, hemoglobin; LDH, lactate dehydrogenase; MCH, mean corpuscular hemoglobin; MCHC, mean corpuscular hemoglobin concentration; MCV, mean corpuscular volume; NEFA, non-esterified fatty acids; NK, non-ketosis; RBC, red blood cell; RDW, red cell distribution width; SCK, subclinical ketosis; WBC, white blood cell.

We analyzed whether hematological parameters on the calving date were associated with ketosis. Significant differences in red blood cell (RBC) count, hemoglobin (Hb), MCV, MCH, MCHC, RDW, and WBC, lymphocyte, monocyte, and eosinophil counts were found among the groups (Table 2). RBC, Hb, MCHC, and lymphocyte counts were not associated with the severity of ketosis. However, the group with severe ketosis had significantly higher MCV and MCH and lower RDW and WBC, monocyte, and eosinophil counts; NK had the lowest MCV and MCH, while CK had the highest values of MCV and MCH. NK displayed the highest values of RDW and WBC, monocyte, and eosinophil counts, whereas CK exhibited the lowest values (Fig. 1).

**Table 2:** The complete blood count (CBC) test results of each group.

	NK	SCK	CK	*p* value
**Erythrocyte**				
RBC count, M/µL	6.42 (6.27–6.59)	6.43 (6.21–6.65)	6.01 (5.67–6.35)	0.023
HCT, %	33.33 ± 0.38	34.69 ± 0.56	34.15 ± 0.93	0.076
Hb, g/dL	11.07 ± 0.12	11.62 ± 0.17	11.15 ± 0.27	0.042
MCV, fL	52.09 (50.97–53.21)	54.19 (52.46–55.92)	56.94 (55.30–58.59)	< 0.001
MCH, pg	17.29 (17.00–17.59)	18.15 (17.65–18.65)	18.63 (18.23–19.04)	< 0.001
MCHC, g/dL	33.24 (33.00–33.48)	33.62 (33.31–33.94)	32.76 (32.35–33.16)	0.003
RDW, %	25.71 (25.05–26.37)	25.20 (24.40–26.00)	23.78 (23.02–24.54)	0.002
Reticulocyte, K/µL	1.60 (1.27–1.92)	2.00 (1.42–2.58)	1.94 (1.41–2.47)	0.339
**Leukocyte**				
WBC count, K/µL	13.20 (12.30–14.10)	12.29 (11.13–13.44)	10.37 (8.74–11.99)	0.004
Neutrophil, K/µL	3.23 ± 0.44	2.48 ± 0.56	2.76 ± 0.57	0.449
Lymphocyte, K/µL	7.55 ± 0.49	7.79 ± 0.59	5.94 ± 0.76	0.013
Monocyte, K/µL	2.19 (2.00–2.37)	1.84 (1.60–2.08)	1.54 (1.32–1.76)	< 0.001
Eosinophil, K/µL	0.23 ± 0.03	0.17 ± 0.02	0.12 ± 0.02	0.009
Basophil, K/µL	0.005 ± 0.001	0.003 ± 0.001	0.004 ± 0.001	0.311
**Platelet**				
PLT count, K/µL	329.24 ± 11.38	328.39 ± 12.13	302.18 ± 14.87	0.286
MPV, fL	6.51 ± 0.06	6.75 ± 0.09	6.75 ± 0.10	0.051
PDW, fL	7.84 ± 0.12	8.08 ± 0.12	8.00 ± 0.15	0.214
PCT, %	0.21 ± 0.01	0.22 ± 0.01	0.20 ± 0.01	0.250

Data obtained using one-way ANOVA are expressed as mean (95% confidence interval), and data obtained using the Kruskal–Wallis test are expressed as mean ± standard error of the mean.

RBC, red blood cell; HCT, hematocrit; Hb, hemoglobin; MCV, mean corpuscular volume; MCH, mean corpuscular hemoglobin; MCHC, mean corpuscular hemoglobin concentration; RDW, red cell distribution width; WBC, white blood cell; PLT, platelet; MPV, mean platelet volume; PDW, platelet distribution width; PCT, plateletcrit.

### Association between ketosis and serum biochemistry

Serum biochemical and mineral parameters on the calving date were analyzed to determine their association with ketosis. BHBA showed significant differences among groups on the calving date despite the small gap of value. There were significant differences in NEFA, total cholesterol (TC), TB, creatinine, alanine transaminase (ALT), lactate dehydrogenase (LDH), and amylase among groups. TC and creatinine levels failed to demonstrate a relationship with the severity of ketosis, but NEFA and TB had a positive relationship with the severity of ketosis. CK had the highest concentrations of NEFA and TB, while NK had the lowest. However, ALT, LDH, and amylase had negative relationships with the severity of ketosis; these parameters were higher in NK, SCK, and CK in this order. ALB exhibited a negative relationship with the severity with weak significance (*p* = 0.051), and the NK group had a significantly higher ALB level than the CK group (Table 3 and Fig. 1).

**Table 3:** Serum biochemistry and mineral levels by each group.

	NK	SCK	CK	*p* value
BHBA, mmol/L	0.50 ± 0.02	0.62 ± 0.04	0.68 ± 0.06	0.009
Glucose, mg/dL	68.37 ± 1.58	69.88 ± 2.37	73.79 ± 3.65	0.783
NEFA, uEq/L	452.08 ± 23.38	621.85 ± 53.28	902.31 ± 87.87	< 0.001
TG, U/L	3.87 ± 0.25	3.35 ± 0.27	2.93 ± 0.16	0.100
TC, mg/dL	83.21 ± 2.05	83.26 ± 3.19	74.21 ± 3.15	0.037
TP, g/dL	6.74 (6.52–6.96)	6.83 (6.55–7.11)	6.85 (6.58–7.13)	0.787
Albumin, g/dL	3.84 (3.74–3.93)	3.74 (3.65–3.83)	3.66 (3.52–3.79)	0.051
Globulin, g/dL	2.90 (2.67–3.14)	3.09 (2.81–3.37)	3.20 (2.88–3.51)	0.277
TB, U/L	0.39 ± 0.02	0.54 ± 0.04	0.70 ± 0.07	< 0.001
BUN, mg/dL	16.06 (15.06–17.06)	15.81 (14.24–17.39)	15.87 (14.27–17.46)	0.955
Creatinine, mg/dL	1.44 (1.39–1.49)	1.56 (1.48–1.63)	1.53 (1.44–1.61)	0.031
ALT, U/L	19.92 (18.46–21.38)	17.06 (15.02–19.10)	14.79 (13.18–16.40)	< 0.001
AST, U/L	86.21 ± 2.66	80.26 ± 2.40	81.62 ± 5.42	0.099
ALP, U/L	230.30 (209.62–250.97)	207.06 (178.33–235.79)	205.10 (177.76–232.44)	0.238
GGT, U/L	23.58 (21.07–26.09)	23.48 (20.48–26.49)	22.31 (20.08–24.54)	0.804
LDH, U/L	1030.44 ± 19.34	976.56 ± 28.35	926.79 ± 26.84	0.002
Amylase, U/L	25.51 (22.57–28.45)	22.73 (19.40–26.07)	19.44 (15.36–23.53)	0.046
Lipase, U/L	83.17 ± 6.84	70.16 ± 5.62	87.04 ± 13.28	0.880
Calcium, mg/dL	8.10 ± 0.14	7.83 ± 0.27	7.86 ± 0.24	0.449
Mg, mg/dL	2.33 ± 0.05	2.49 ± 0.07	2.53 ± 0.10	0.092
P, mg/dL	5.09 ± 0.12	5.02 ± 0.29	4.62 ± 0.29	0.187

Data obtained using one-way ANOVA are expressed as mean (95% confidence interval), and data obtained using the Kruskal–Wallis test are expressed as mean ± standard error of the mean.

BHBA, β-hydroxybutyrate; NEFA, non-esterified fatty acids; TG, triglyceride; TC, total cholesterol; TP, total protein; TB, total bilirubin; ALT, alanine transaminase; AST, aspartate transaminase; ALP alkaline, phosphatase; GGT, γ-glutamyl transferase; LDH, lactate dehydrogenase; BUN, blood urea nitrogen; Mg, magnesium; P, inorganic phosphorus.

## Discussion

The present study aimed to predict whether Holstein Friesian cows are at risk of ketosis during the transition period by analyzing hematological and serum biochemical parameters on the calving date. To the best of our knowledge, this study is the first to identify the associations of hematological and serum biochemical parameters on the calving date with ketosis during the postpartum transition period. We observed that elevated MCV, MCH, NEFA and TB were positively associated with ketosis and its severity. Cows with higher RDW, WBC count, monocyte count, eosinophil count, ALB, ALT, LDH, and amylase were at lower risk of ketosis and its severity. MCV, MCHC, lymphocyte count, and eosinophil count were significantly different between subclinical and clinical ketosis.

RBC indices are associated with body dysfunction. However, only a few previous studies investigated the association between ketosis and RBC indices in cows showing no association between ketosis and MCV, MCH, MCHC, and RDW^13,25^; a finding is different from that in the current study. As shown in Table 1, the incidence of ketosis varied during the transition period. The discrepancy between our findings and previous ones may stem from differences in study design, such as monitoring frequency. In the present study, elevated MCV and MCH were associated with ketosis. Humans with liver disease and deficiencies in vitamin B_9_ or B_12_ showed elevated MCV and MCH^26,27^. The liver is an organ closely related to ketosis. Dairy cows with elevated MCV and MCH might have a less functional liver, which may lead to ketosis. RDW is known to be elevated in patients with liver disease. Interestingly, the RDW findings in this study are inconsistent with those in previous studies on liver diseases; the reason is unclear, but a previous study showed a similar though not significant association between RDW and the severity of ketosis in sheep^28^. Our findings suggest that unlike cows with other liver diseases, those with severer ketosis have lower RDW values.

Accumulating studies indicate that ketosis is associated with decreased WBC count, and that ketosis increases the risk of diseases. Dairy cows with ketosis show decreased WBC count^14,29^. Human adults with ketogenic diets show a significant decrease in WBC count^30^. Ketosis increases the risk of metritis and mastitis^31^. Consistently, the present study found lower WBC counts and severer ketosis during the transition period. The decrease in WBC count in cows with ketosis might contribute to the susceptibility to infectious diseases. NK cows had extremely significantly more monocytes than CK cows and significantly more monocytes than SCK cows. Both cows and humans with hyperketonemia have lower monocyte counts^32,33^. Humans in the low-energy state have a reduced number of circulating monocytes migrated from bone marrow as a result of signaling from hepatocyte^34^. The liver might already play a role in recruiting a smaller population of monocytes from the bone marrow before ketosis in the ketosis groups. In addition, NK cows showed very significantly more eosinophils than SCK cows, while SCK cows had significantly more eosinophils than CK cows. Humans with ketosis also show lower eosinophil counts^35,36^. It is speculated that eosinophil counts are lowered by oxidative stress, elevated apoptosis, and the inhibition of cell proliferation^35^.

As for serum biochemistry, most of the parameters associated with ketosis (NEFA, ALB, TB, ALT, and amylase) are highly related to hepatic dysfunction. NEB in animals promotes lipolysis, which mobilizes NEFA from the adipose tissue to most body tissues through the blood stream. The liver is the main organ to process NEFA into three forms: ATP via complete oxidation, ketone synthesis via incomplete oxidation, or very low-density lipoproteins via re-esterification^4,37^. The liver of cows with ketosis may not be able to remove NEFA around parturition even before ketosis onset, compared to non-ketosis. In addition, ALB and TB are commonly used to indicate hepatic insufficiency, and our results on ALB and TB correspond with previous findings. Lower serum ALB levels were found in CK, SCK, and NK in this order, indicating decreased liver function^21,38^. The level of TB increases at ketosis, indicating liver insufficiency^18,39^. Increased TB levels at parturition also suggest decreased liver function. Notably, in the current study CK cows had the lowest level of ALT while NK cows had the highest on the calving day. Previous studies reported that ALT was insensitive in ruminants including cows^21,40,41^. However, elevated hepatic apoptosis and a high ALT level were observed in dairy cows with ketosis^42^. Our findings in this study differ from the previous results. In dogs and cats, low ALT levels indicate hepatic atrophy, while high ALT levels indicate hepatocellular injury or leakage^43^. Further studies are required to investigate the association between serum ALT levels on the calving date and ketosis.

To the best of our knowledge, no study has reported an association between serum amylase and ketosis in cattle. However, low concentrations of serum amylase are associated with the degree of liver dysfunction, such as non-alcoholic fatty liver, metabolic syndrome, and insulin resistance^44,45^. In human medicine, a previous study revealed that low serum amylase levels were inversely related to serum ketone bodies^46^. Insulin resistance is also associated with ketosis in dairy cows^47^. Our findings suggest that ketosis may be associated with low serum amylase concentration.

LDH is known to contribute to glycolysis, and elevated LDH levels are a marker of organ damage, including muscles and liver damage^48^. LDH was expectedly higher in cows with ketosis because other biomarkers indicated obvious damage to the liver. However, we found that LDH had a negative relationship with the severity of ketosis. Information on the association between LDH and ketosis is limited. Energy production might presumably be related to our unexpected findings that NK had the highest LDH level and CK had the lowest. Indeed, LDH plays an important role in the regulation of glycolysis^49^. Lower LDH levels in ketosis might indicate a decreased ability to use glucose for energy, which might lead to fat mobilization.

This study has some limitations. First, it was conducted at only one farm. More dairy cows at different farms in various regions should have been investigated. Furthermore, parity and season affect blood parameters in cattle^50–53^. The incidence of ketosis is associated with parity and season^5–11^. The number of dairy cows used in the study was insufficient for statistical analysis by parity and season. Therefore, we could not investigate the variations of blood parameters by parity and season, according to the incidence of ketosis. In addition, other factors like biological variability might influence dairy cows even though we made efforts to exclude factors irrelevant to ketosis. However, the current study demonstrated that the parameters of the complete blood count (CBC) test and serum biochemistry on their own on the calving date were associated with ketosis and its severity. Our findings suggest that theses parameters may be predictive indicators for ketosis. Therefore, it may be more helpful to predict Holstein cows susceptible to ketosis using these parameters along with other risk factors previously reported.

In summary, the present study found that hematological and serum biochemical parameters on the calving date were associated with ketosis and its severity during the postpartum transition period. Dairy Holstein cows with clinical ketosis showed significantly the highest values of MCV, MCH, NEFA, and TB but significantly lowest values of RDW; WBC, monocyte, and eosinophil counts; and ALB, ALT, LDH, and amylase levels. Dairy Holstein cows with non-ketosis showed the opposite results. Our findings suggest that hematological and serum biochemical parameters can help select dairy Holstein cows susceptible to ketosis. However, further studies are required to elucidated the actual prognostic application using these parameters.

## Materials and methods

### Animals

A total of 126 Holstein Friesian cows were used in this study. The cows were raised and calved at one farm of the National Institute of Animal Science, in Cheonan, Republic of Korea. The animals calved from January 2018 to January 2021 and were normally milked during the transition period. The animals were fed the same total mixed rations ad libitum, which were composed of concentrates, soybean meal, corn silage, alfalfa hay, timothy hay, enzyme, minerals, and vitamin additives. They were milked twice a day in the morning and evening. Dairy cows milked less than twice a day or receiving any medication or nutritional or microbial supplement capsules and boluses after calving were excluded.

### Blood sampling and case definition

Blood sampling was conducted once every 3 days (8 times in 21 days from the calving date) during the postpartum transition period. Blood was sampled once from the jugular vein of the cows in 6–23 h postpartum on the calving date and seven times in the morning from day 3 postpartum when the cows began to be fed as soon as they finished milking. Blood was collected using ethylenediaminetetraacetic acid (EDTA) and serum-separating tube (SST) tubes. BHBA was measured with electronic handheld meters (FreeStyle Optium Neo, Abbott Diabetes Care Ltd., Witney, UK) and β-ketone test strips (FreeStyle Optium H β-ketone, Abbott Diabetes Care Ltd., Witney, UK) like in previous studies^54–57^, immediately after blood sampling. For more accuracy, blood BHBA was randomly measured more than once, and the results were either the same or differed by ≤ 0.1 mmol/L, and none of the cases belonged to another group. In addition, the device (Freestyle Optium Neo) was changed monthly, and cross-checks were done with another handheld meter (Optium Xceed, Abbott Diabetes Care Ltd., Witney, UK) weekly, which offered quality control strips.

The cows were divided into three groups according to the highest BHBA concentration in eight samples during the post-calving transition period: a non-ketosis group (< 1.2 mmol/l, n = 63), a subclinical ketosis group (1.2 ≤ BHBA ≤ 2.9 mmol/l, n = 34), and a clinical ketosis group (≥ 3.0 mmol/l, n = 29)^10,56^.

### Blood analyses and data collection

Blood analyses (hematology and serum biochemistry) were performed at a laboratory of the National Institute of Animal Science, located close to the farm where blood sampling was conducted. The CBC test was performed with a hematology analyzer (Procyte Dx^®^ hematology analyzer, IDEXX Laboratories, Westbrook, MA, USA) with blood collected in EDTA tubes within 1 h after blood collection. Monthly quality control assessments were conducted for the hematology analyzer. The profiles of the CBC test consisted of three types: erythrocytes, leukocytes, and platelets. The erythrocyte parameters included RBC count, hematocrit (HCT), Hb, MCV, MCH, MCHC, RDW, and reticulocyte count. The leukocyte parameters included WBC, neutrophil, lymphocyte, monocyte, eosinophil, and basophil counts. The platelet parameters included platelet (PLT) count, mean platelet volume (MPV), platelet distribution width (PDW), and plateletcrit (PCT).

Serum was harvested by centrifuging SST tubes at 3000 rpm for 10 min. Serum biochemistry and mineral analyses were conducted using two biochemistry automatic analyzers (Catalyst™ Dx chemistry analyzer, IDEXX Laboratories, Westbrook, MA, USA and Hitachi 7180, Hitachi Ltd., Tokyo, Japan). TB, amylase, and lipase were analyzed immediately after the serum was harvested using a Catalyst™ Dx chemistry analyzer, which had quality control assessments monthly. Glucose, NEFA, TG, TC, total protein (TP), ALB, BUN, creatinine, ALT, aspartate transaminase (AST), alkaline phosphatase (ALP), gamma-glutamyl transferase (GGT), LDH, calcium, magnesium, and inorganic phosphorus were measured using a Hitachi 7180 after calibration and quality control assessments with commercial enzyme assay kits from Wako (Fujifilm Wako Pure Chemical Ltd., Osaka, Japan). Globulin was calculated by subtracting ALB from TP. The serum was frozen and stored at − 70 °C pending analysis using a Hitachi 7180, and serum biochemical analysis was conducted alongside in a single day.

### Statistical analyses

Statistical analyses were performed using SPSS software (version 26.0; IBM Corp., Armonk, NY, USA). The Shapiro–Wilk test and Levene’s test were used for normality analysis and equality of variances for one-way analysis of variance (ANOVA). The Kruskal–Wallis test was used for parameters that did not satisfy normality analysis nor equality of variances. The difference analysis among groups was conducted with post-hoc Tukey honestly significant difference for One-way ANOVA and Mann–Whitney U test with Bonferroni’s method for the Kruskal–Wallis test. One-way ANOVA was used to evaluate RBC count, MCV, MCH, MCHC, RDW, reticulocyte count, WBC count, Monocyte count, TP, ALB, globulin, BUN, creatinine, ALT, ALP, GGT, and amylase. The Kruskal–Wallis test was applied to analyze HCT, Hb, neutrophil count, lymphocyte count, eosinophil count, basophil count, PLT count, MPV, PDW, PCT, BHBA, glucose, NEFA, TG, TC, TB, AST, LDH, lipase, Ca, Mg, and P. Data obtained using one-way ANOVA are expressed as mean (95% confidence interval), whereas data obtained using the Kruskal–Wallis test are expressed as mean ± standard error of the mean.

Significance levels were interpreted based on *P*-values as follows: 0.01 ≤ *p* < 0.05, significant; 0.001 ≤ *p* < 0.01, very significant; and *p* < 0.001, extremely significant. Significance levels using Mann–Whitney U test with Bonferroni’s test were divided into the following three categories: 0.003 ≤ *p* < 0.017, significant; 0.0003 ≤ *p* < 0.003, very significant; and *p* < 0.0003, extremely significant.

### Ethical approval

This research was approved by the Institutional Animal Care and Use Committee (IACUC) at the National Institute of Animal Science, the Republic of Korea (approved number: NIAS-2020127). All experimental procedures involving animals were conducted in strict accordance with relevant guidelines and regulations. All methods used for in vivo studies in cows were in accordance with ARRIVE guidelines.

## Data Availability

The dataset analyzed for the current study is available from the authors upon reasonable request.
